# IL-6/ERK signaling pathway participates in type I IFN-programmed, unconventional M2-like macrophage polarization

**DOI:** 10.1038/s41598-022-23721-9

**Published:** 2023-02-01

**Authors:** Limin Yang, Panpan Guo, Pei Wang, Wei Wang, Jianghuai Liu

**Affiliations:** 1 State Key Laboratory of Pharmaceutical Biotechnology and MOE Key Laboratory of Model Animals for Disease Study, Model Animal Research Center at Medical School of Nanjing University, Nanjing, 210061 China; 2 Yancheng First Hospital, Affiliated Hospital of Nanjing University Medical School, Yancheng, 224006 China; 3 Department of Rheumatology and Immunology, The Affiliated Drum Tower Hospital of Nanjing University Medical School, Nanjing, 210008 China; 4 The First People’s Hospital of Yancheng, Yancheng, 224006 China; 5 Jiangsu Key Laboratory of Molecular Medicine, Medical School of Nanjing University, Nanjing, 210093 China

**Keywords:** Interferons, Interleukins, Monocytes and macrophages, Cancer immunotherapy, Cytokines, Signal transduction

## Abstract

Type I interferons (IFN-Is) have been harnessed for cancer therapies due to their immunostimulatory functions. However, certain tumor-tolerating activities by IFN-Is also exist, and may potentially thwart their therapeutic effects. In this respect, our previous studies have demonstrated a monocyte-orchestrated, IFN-I-to-IL-4 cytokine axis, which can subsequently drive M2-skewed pro-tumoral polarization of macrophages. Whether other IFN-dependent signals may also contribute to such an unconventional circumstance of M2-like macrophage skewing remain unexplored. Herein, we first unveil IL-6 as another ligand that participates in IFN-dependent induction of a typical M2 marker (ARG1) in transitional monocytes. Indeed, IL-6 significantly promotes IL-4-dependent induction of a major group of prominent M2 markers in mouse bone marrow-derived macrophages (BMDMs) and human peripheral blood-derived macrophages, while it alone does not engage marked increases of these markers. Such a pattern of regulation is confirmed globally by RNAseq analyses in BMDMs, which in turn suggests an association of IL-6-amplified subset of M2 genes with the ERK1/2 signaling pathway. Interestingly, pharmacological experiments establish the role of SHP2-ERK cascade in mediating IL-6’s enhancement effect on these M2 targets. Similar approaches also validate the involvement of IL-6/ERK signaling in promoting the IFN-dependent, unconventional M2-skewing phenotype in transitional monocytes. Furthermore, an inhibitor of ERK signaling cooperates with an IFN-I inducer to enable a greater antitumor effect, which correlates with suppression of treatment-elicited ARG1. The present work establishes a role of IL-6/ERK signaling in promoting M2-like macrophage polarization, and suggests this axis as a potential therapeutic target for combination with IFN-I-based cancer treatments.

## Introduction

Recent advances in cancer research have demonstrated the possibility of harnessing antitumor immunity for effective treatment of cancer^1,2^. Such progresses establish the main therapeutic actions by the T cells from the adaptive immune system against the malignant cells. On the other hand, since the function of adaptive immunity requires the priming, amplification and actuation by the innate immune system, the proper engagement of the innate arm of immunity also hold great potential for activation and enhancement of tumor immune-targeting^3^.

Activation of many pattern-recognition innate immune pathways has been experimented for antitumor activities. The existing evidence demonstrates that engagement of anti-viral innate immune signaling pathways, i.e., the nucleic acid-sensing pathways, can often result in significant antitumor effects^4,5^. Triggered via various membrane-bound or cytosolic nucleic acid receptors, these different pathways converge on the production of type I IFNs (IFN-I). Subsequently, IFN-I signaling can promote antigen presentation in dendritic cells and elevate the effector function of T/NK cells, which coordinately contributes to enhancing anti-tumor immunity^6,7^. Despite the critical role of IFN-I in innate immune activation against cancer, some studies have revealed certain pro-tumoral effects by IFN-I in given contexts^8,9^. Therefore, in-depth understandings to the latter aspect of IFN-I function may contribute to more optimized strategies to exploit the innate immune system for treatment of cancer.

IFN-Is signal through a common, broadly expressed receptor to drive the formation of an activated, STAT1/STAT2/IRF9 complex, which in turn leads to induction of many interferon-stimulated genes (ISGs)^10,11^. However, the effects of IFN-I in various tumor-associated cell types can significantly differ^9,12^. How such multi-pronged regulation is functionally integrated is not fully understood.

Tumor-associated macrophages (TAMs) constitute the major immune cell population within the tumor microenvironment^13,14^. TAMs are believed to mostly originate from the recruited monocytes from circulation, although some recent studies have also suggested the contributions of tissue-resident macrophages to TAMs^15–17^. Upon recruitment to the tumors from circulation, the monocytes further mature and differentiate into the TAMs^17^. As the phenotypes of monocytes/macrophages are highly influenced by the environmental cues, the TAMs exhibit remarkable functional heterogeneity^15,18^. Although the early classification of macrophage phenotypes into M1/M2 dichotomous states is certainly an over-simplification^19^, it provides an entry point for dissecting their functional complexity^20^. Previously, via studying the role of IFN-I in the tumor-associated monocytes and macrophages, we have established an “unconventional” aspect by IFN-I to trigger monocyte production of IL-4, which in turn drives the mature macrophages toward M2-skewed phenotypes in vitro and in tumors^21,22^. Indeed, such monocytes/macrophages-involved regulatory axis thwarts IFN-inducer-mediated antitumor effects in mice. However, considering the multitude of cytokines that may be produced by monocytes^23,24^, it remains possible that other IFN-dependent factors from monocytes may also contribute to driving such an unconventional M2-like macrophage polarization program.

In the present study, we demonstrate that IFN-I additionally trigger monocytes to produce IL-6, which cooperates with IL-4 to shape the M2-skewed macrophage phenotype. Our results further show a critical role by IL-6/ERK signaling to promote IL-4-dependent macrophage functional skewing. Our work therefore suggests IL-6/ERK pathway as a potential target for enhancement of IFN-I-involved anti-tumor therapies.

## Results

### IFN-stimulated transitional monocytes release IL-6 to promote IL-4-mediated induction of ARG1 in macrophages

**Figure 1:**
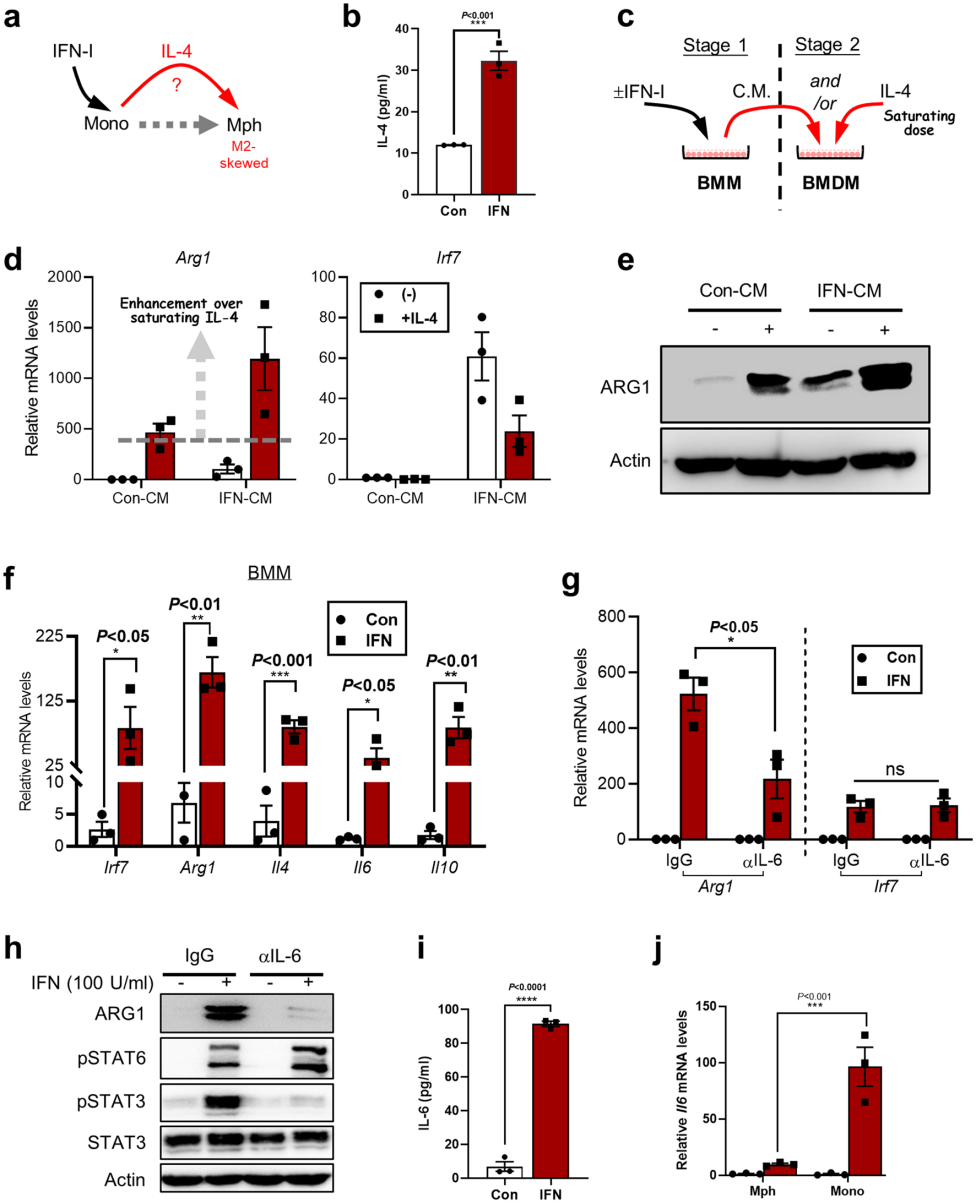
IFN-stimulated transitional monocytes release IL-6 to promote IL-4-mediated induction of ARG1 in macrophages. (**a**) A schematic drawing shows the hypothesis that IFN-treatment of monocytes may release of a yet-to-be determined soluble factor(s) that acts together with previously established IL-4 to drive the M2-skewing of mature macrophages. The differentiation transition from monocytes to macrophages is also denoted by a dotted arrow. (**b**) BM mononuclear cells were treated with IFN (100 U/ml) for 24 h in the presence of M-CSF (20 ng/ml), the production of IL-4 in cell supernatants was determined by ELISA (n = 3, ± SEM). (**c–e**) The experiment scheme is shown in (**c**). In the first stage, BM mononuclear cells (BMM) were treated with M-SCF ± IFN for 48 h. The conditioned media (C.M.) were harvested. In the second stage, the C.M. from control or treated cells were transferred to naïve, mature macrophages (BMDMs). The BMDMs were treated for 48 h with either the C.M. alone or the C.M. and a saturating dose of IL-4. The cells were harvested and subjected to qPCR (n = 3, ± SEM) (**d**) or Western blot (WB) analyses (**e**). (**f**) BM mononuclear cells were treated with M-SCF ± IFN for 48 h, samples were harvested for mRNA analyses of indicated markers and cytokine genes (n = 3, ± SEM). (**g,h**) BM mononuclear cells were treated with M-CSF ± IFNβ for 48 h. In some groups, neutralizing Abs against IL-6 (5 μg/ml) were added to the medium as indicated. Samples were harvested after 48 h for mRNA [n = 3, ± SEM] (**g**) or protein (**h**) analyses. (**i**) BM mononuclear cells were treated with IFN for 24 h in the presence of M-CSF, the production of IL-6 in cell supernatants was determined by ELISA (n = 3, ± SEM). (**j**) BM mononuclear cells were treated with M-SCF ± IFN for 48 h, monocytes (Ly6C^+^F4/80^−^) and macrophages (F4/80^+^) were sorted by flow cytometry. Samples from sorted cells were subjected to qPCR analysis (n = 3, ± SEM). For most statistical analyses related to this figure, unpaired t tests were performed (*P* value marked on the graph). For (**j**), 2-way ANOVA tests were performed (the adjusted *P* value presented). Asterisks correspond to *P* values (****P < 0.0001; ***P < 0.001; **P < 0.01; *P < 0.05; *ns* not significant).

We previously unveiled a monocyte-orchestrated IFN-I-to-IL-4 cytokine axis poised to subsequently instigate macrophages toward a pro-tumoral phenotype^22^. This particular regulatory program may be modeled in vitro in IFN-treated bone marrow (BM) mononuclear cells instructed to differentiate toward macrophages [by M-CSF]^21,22^. In this system referred to as the “transitional monocytes”, it has been demonstrated that the IFN-triggered IL-4 from the monocytes in turn acts on their differentiated macrophage progenies to drive the expression of a number of prominent M2 markers (Fig. 1a), including the commonly used arginase 1 (ARG1)^22^. Here, using cells from previously described *Arg1*-YFP knock-in mice^22^, we added evidence that the IFN-to-ARG1 axis and the associated pSTAT6 signaling in the transitional monocytes could be abrogated by an antibody against IL-4 (Supplementary Fig. 1a). Therefore, these confirmatory results (extending those derived from WT or IL-4RA-deficient cells^22^) firmly establish that IL-4 is indeed necessary for IFN-induced, unconventional M2-like program in transitional monocytes. We additionally showed via ELISA analyses that IFN-I induced an approximately threefold increase of IL-4 release from transitional monocytes [reaching ~ 30 pg/ml in bulk medium] (Fig. 1b).

Despite the essential role of IL-4 in IFN-elicited M2-skewing of transitional monocytes, considering the myriad of known monocyte-produced factors under diverse conditions^23,24^, it is possible that other IFN-dependent signals could also contribute to promoting macrophage M2-skewing. To assay such an effect, we considered to mask the induced IL-4 release by using exogenous IL-4 at a saturating concentration. Different concentration of IL-4 was titrated on the mature bone marrow-derived macrophages (BMDMs) with the *Arg1*-YFP genotype. The expression levels of YFP (led by an IRES, therefore indirectly indicating *Arg1* mRNA), as well as those of ARG1 protein were analyzed (Supplementary Fig. 1b–d). Based on the analyses, we later chose to use IL-4 at a saturating concentration of 100 ng/ml. The effects by the conditioned medium (CM) from the control or IFN-stimulated transitional monocytes (Con- or IFN-CM) combined with ± IL-4 on BMDMs were examined (Fig. 1c). As expected, the IFN-CM alone contained activity to induce higher *Arg1* mRNA and protein expression compared to the Con-CM (Fig. 1d,e). Importantly, the IFN-CM could also enhance *Arg1* induction when combined with the saturating dose of IL-4 (Fig. 1d,e). This strongly suggested that some other soluble factors from the IFN-CM could cooperate with IL-4 to drive *Arg1* expression.

Subsequently, we further analyzed IFN-stimulated transitional monocytes for the expression of other M2-associated cytokines. Interestingly, we found that the mRNA levels of IL-6 and IL-10 were induced along with that of IL-4 (Fig. 1f). Indeed, both IL-6 and IL-10 have been previously demonstrated to enhance IL-4-dependent induction of macrophage M2 phenotype^25–27^. Next, neutralizing antibodies against IL-6 or IL-10 were applied on IFN-stimulated transitional monocytes to dissect the potential involvement for these cytokines in *Arg1* induction. The results showed that blockade of IL-6, but not IL-10, could noticeably reduce IFN-triggered *Arg1* induction in transition monocytes (Fig. 1g, Supplementary Fig. 1e). A similar effect by anti-IL-6 on ARG1 protein was observed (Fig. 1h). As both IL-6 and IL-10 are poised to activate STAT3, it is worth noting that the pSTAT3 [referring to the Y705-phophorylated species in this study] induction in IFN-treated transitional monocytes was also largely suppressed by anti-IL-6, correlating with the effect on ARG1 induction (Fig. 1h). Indeed, the levels of IL-6 were significantly elevated in the supernatants of IFN-stimulated transition monocytes [reaching ~ 120 pg/ml] (Fig. 1i).

To analyze the cell compartment responsible for IFN-dependent IL-6 induction, the monocytes (Ly6C^+^F4/80^−^) and macrophages (F4/80^+^) in IFN-stimulated transitional monocytes were sorted out by flow cytometry (Supplementary Fig. 1f, upper). As expected from our previous study, the *Il4* mRNA was only induced in the monocytes, but not in the macrophages (Supplementary Fig. 1f, lower). In a similar pattern, *Il6* was induced far more robustly in the monocyte than the macrophage compartment (Fig. 1j). As we have established that ARG1 induction instead occurs in an F4/80^+^ macrophage-restricted pattern^22^, these results of compartmentalized *Il6* induction points to a model where the monocyte-derived IL-4 and IL-6 act in concert upon the mature macrophages to enforce their M2-skewing.

### IL-6 signaling can tightly coordinate with IL-4 signaling, leading to enhancement of a group of prominent M2 markers in macrophages

**Figure 2:**
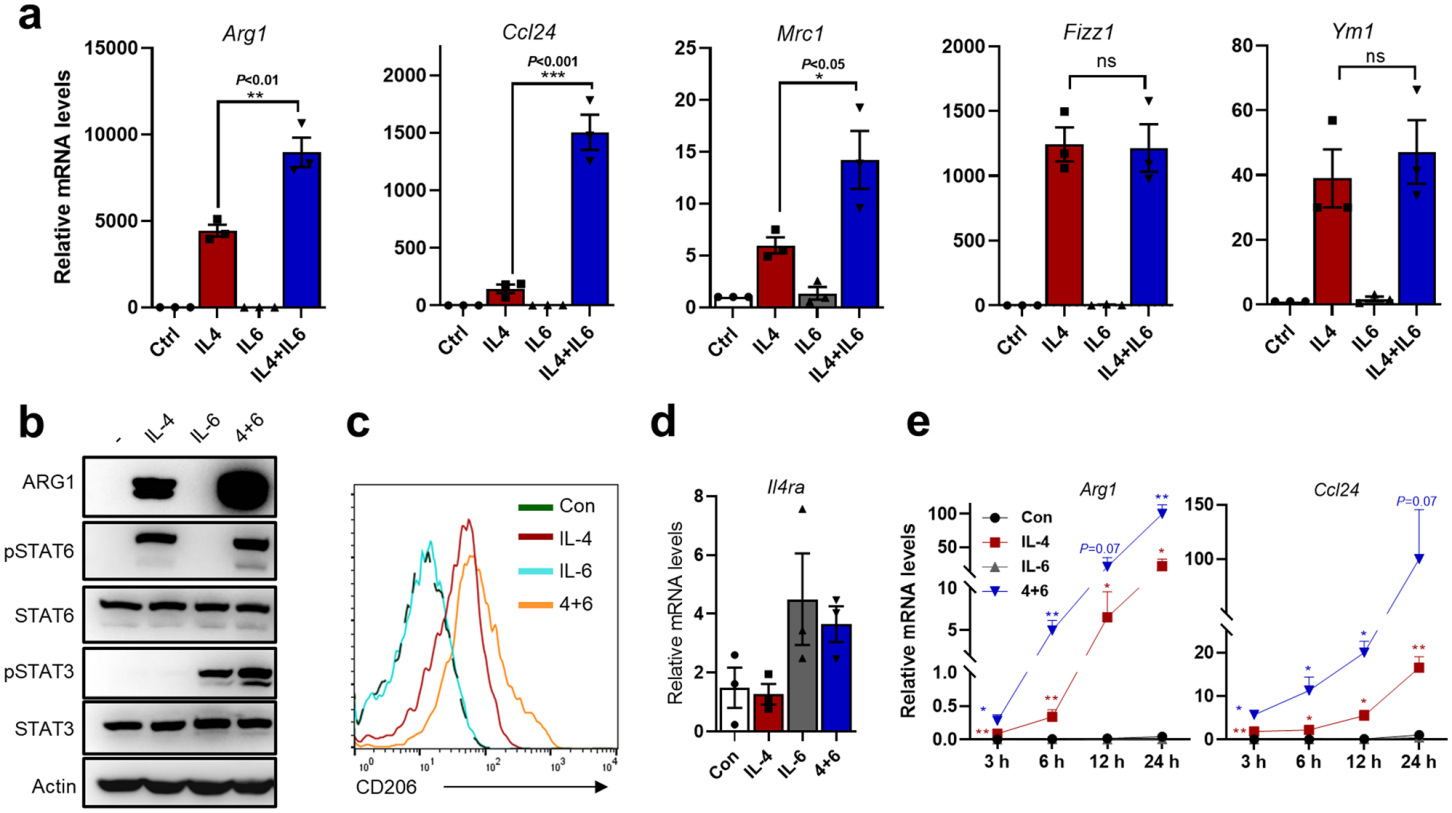
IL-6 signaling can tightly coordinate with IL-4 signaling, leading to enhancement of a group of prominent M2 markers in macrophages. (**a–d**) BMDMs were stimulated with individual cytokines (10 ng/ml of IL-4 or 40 ng/ml of IL-6) or their combination for 24 h. Some samples were subjected to qPCR (n = 3, ± SEM) (**a,d**) and WB analyses (**b**). For some other samples, the levels of intracellular CD206 in BMDMs were analyzed by flow cytometry (**c**). (**e**) Time course expression levels of *Arg1* and *Ccl24* in BMDMs treated with ± IL-4 ± IL-6 were analyzed by qPCR (n = 3, ± SEM). Unpaired t tests were performed for individual markers in (**a**), with *P* values marked on the graphs. For time series of IL-4/IL-6 treatments in (**e**), multiple unpaired t tests (using Holm–Šídák method) were performed to compare the IL-4 group with control group [red asterisks], and the co-treatment group with IL-4 group [blue asterisks], respectively. Asterisks in this figure correspond to *P* values (***P < 0.001; **P < 0.01; *P < 0.05; *ns* not significant).

Unlike the anti-IL-4 treatment that abrogated IFN-dependent ARG1 in transitional monocytes (see Supplementary Fig. 1a), the inhibitory effect by IL-6 blockade on such IFN-ARG1 axis was incomplete (see Fig. 1g,h), pointing to a promotive (rather than determinative) role by IL-6 signaling on macrophage M2-skewing. To further analyze the combinatorial effect of IL-4 and IL-6 on macrophages, we next performed exogenous cytokine treatment experiments on mature BMDMs and analyzed several typical M2 macrophage markers. Largely in correlation with our observations from the IL-6 blockade experiments (see Fig. 1g,h), exogenous IL-6 notably enhanced the induction of *Arg1*, *Ccl24* and *Mrc1* (encoding CD206) by IL-4, while it alone did not act as an effective inducer of these genes (Fig. 2a). Such effects by IL-6 were also consistent with previous investigations^25,27^. The patterns of ARG1 and CD206 protein levels resembled those of their mRNAs (Fig. 2b,c). As IL-13 can function similarly as IL-4 in eliciting the STAT6-dependent, typical M2 macrophage program^28,29^, the effects by IL-6 on IL-4- and IL-13-driven *Arg1* in BMDMs (*Arg1*-YFP) were next examined in parallel. Although IL-4 and IL-13 showed different M2-polarizing potencies, IL-6 indeed engaged a similar pattern of enhancement on induction of *Arg1*/YFP by these two cytokines (Supplementary Fig. 1a,b).

One potential mechanism underlying IL-6’s enhancement of macrophage M2 (IL-4) polarization was the previously suggested IL-6-STAT3-IL-4RA axis^25^. This model would predict an IL-6-engaged global increase of IL-4 signaling and responses. However, although the IL-6-driven up-regulation of *Il4ra* mRNA could be reproduced in our experiments (Fig. 2d), for *Fizz1* and *Ym1*, other two commonly-regarded M2 macrophage markers, co-addition of IL-6 did not enhance their induction by IL-4 (Fig. 2a). Furthermore, IL-4-stimulated pSTAT6 levels were also unaltered by IL-6 co-addition (Fig. 2b). In the nuclear fraction of the cells, the IL-4-dependent pSTAT6 levels were also unresponsive to IL-6 treatment (Supplementary Fig. 2c). Conversely, IL-4 did not apparently affect the levels of IL-6-stimulated pSTAT3 in total cell lysates and nuclear extracts (Fig. 2b, Supplementary Fig. 2c). Collectively, these results suggest an alternative mode of action by IL-6, where its signaling may be more intricately integrated with that of IL-4 in macrophages.

Next, we examined the time course on *Arg1* and *Ccl24* induction by the singular or combinatory treatments with IL-4/IL-6. Interestingly, the IL-6-enhanced *Arg1* and *Ccl24* mRNA induction by IL-4 became apparent as early as 3 h, and was observed consistently throughout the time points analyzed (6, 12, and 24 h following the treatment). Such kinetics of the IL-6-mediated enhancement effect is generally in line with those for induction of target genes by IL-4 alone (Fig. 2e). These results support that IL-6-dependent increase on induction of such M2 markers in BMDMs can be attributed to tightly coordinated interactions between IL-4 and IL-6 signaling. This model is different from the previous one centered on the secondary response by IL-6-engaged IL-4RA upregulation^25,27^, which would shape a “delayed” enhancement effect.

A pre-treatment experiment was additionally performed to dissect the influences by an IL-6-established functional state or on-going IL-6 signaling on the macrophage responses to IL-4. The BMDMs were pre-treated with ± IL-6 for 24 h followed by medium wash-off. The pre-treatment length of 24-h was chosen for explicit establishment of an IL4RA-upregulated state^25^, while the medium wash-off would promptly dampen on-going IL-6 signaling^30^. These cells were chased with ± IL-4 for another 24 h. After harvesting different groups of cells at the same time, their levels of *Arg1*, *Ccl24* and *Il4ra* mRNA were determined (Supplementary Fig. 2d). Treatment of IL-4 for 24 h was sufficient to induce an M2 program. However, despite the clear persistence of increased *Il4ra* levels in IL-6-pretreated groups, the levels of IL-4-induced *Arg1* and *Ccl24* were not markedly enhanced by such pre-treatment condition (Supplementary Fig. 2d, compare the red and green bars). In contrast, the BMDMs treated concomitantly with IL-4 and IL-6 for 24 h (blue bars) showed a more prominent induction of markers than the IL-4-only group (Supplementary Fig. 2d). These results also suggest a major contribution of an integrated IL-6 and IL-4 signaling, seemingly to prevail over that of an IL-6-primed cell state (e.g., with IL4RA up-regulation), for enhancement of IL-4-driven M2 skewing of BMDMs.

### Co-addition of IL-6 with IL-4 leads to enhanced expression in a major group of IL-4 targets, together with other patterns of regulation of gene expression

To obtain a global view on IL-6-mediated regulation of IL-4 response in macrophages, we performed RNAseq analyses on samples from BMDMs stimulated for 24 h with IL-4 or IL-4/IL-6 combined. Although we did not include the IL-6-only group in our experiments, we noted a previous microarray database [GSE70626] established via single and combinatorial IL-4/IL-6 treatments of BMDMs^31^, and would late use such resource for reference. Using our own dataset, principal component analysis (PCA) showed variable gene expression patterns among different treatment groups (Supplementary Fig. 3a). Differential gene expression analyses (using a cutoff of logFC > 2) revealed that both IL-4 and IL-4/IL-6 co-treatment led to stimulation of expression in hundreds of genes (413 and 577, respectively), of which a significant portion (256) are common to both groups (Supplementary Fig. 3b). Therefore, the IL-4/IL-6-treated cells remain “M2-like”, although the cells in the co-treatment group apparently feature uniquely induced genes (221). Of the genes induced by different treatments, the heat map revealed some further details. Indeed, a major subset of IL-4-induced M2 markers were further enhanced upon co-addition with IL-6 (Supplementary Fig. 3c, see the black boxes [3rd and 5th from the top of the graph]). Nevertheless, the yellow box with the dotted lines indicates some other M2 genes that were not apparently changed upon IL-6 co-addition (Supplementary Fig. 3c). The genes induced by IL-4/IL-6 co-treatment but not by IL-4 only are highlighted by the green box. Interestingly, IL-6 co-addition with IL-4 also apparently led to down-regulation of a significant portion of IL-4-responsive genes (Supplementary Fig. 3c, grey box), a pattern also previously noted by others using microarray^31^. Overall, despite the use of different methodology (RNAseq *vs* microarray), the regulatory patterns of gene by IL-4 alone or IL-4/IL-6 combination were consistent between the current and previous study^31^, which encouraged our further analyses of the present dataset.

As we were interested in IL-6-enhanced subset of M2 genes, genes notably up-regulated by IL-4/IL-6 co-treatment over IL-4 [LogFC > 1.5, 41 genes] were selected (Supplementary Fig. 3d). We defined this group of 41 genes as the “IL-6-enhanced IL-4 targets”. Importantly, using the previous microarray results as a reference to define targets genes by IL-6 alone^31^, only a small fraction of such IL-6 targets are common with our gene list above (13%, 5 out of 39 reference IL-6 target genes with LogFC > 1.5). On the other hand, a larger fraction of “IL-6-enhanced IL-4 targets” deduced from re-analyses of the previous microarray database are common with our gene list (44%, 8 out of 18 reference genes with enhancement levels of more than 1.5 [LogFC]), further validating our assignment of this 41-gene list. Indeed, besides *Arg1*, *Mrc1* and *Ccl24* noted earlier, other well-known M2 marker, including *Chil4* and a number of chemokine genes, are within this IL-6-enhanced group (Supplementary Fig. 3d, annotation in red). The qPCR analyses confirmed such expression patterns for these markers (Supplementary Fig. 3e). Here, IL-6 alone did not significantly induce the expression of these genes (Supplementary Fig. 3e). Therefore, such extended analyses demonstrated that IL-6 could indeed augment IL-4’s inducing activities on a major group (but not all) of its target genes in BMDMs. Although an opposite effect by IL-6 was also established by the transcriptome analyses (see Supplementary Fig. 3c, the grey box), it was beyond the scope of the present study. We noted that the IL-6-enhanced IL-4 targets as a group exhibited a trend of higher expression levels under the IL-4-treated condition, compared to those targets suppressed by IL-6 [close to reaching statistical significance, *P* = 0.064] (Supplementary Fig. 3f).

Our earlier observations showed that the enhancement effect of IL-6 on certain IL-4-engaged targets was likely to involve concomitant signaling by these cytokines (see Fig. 2e, Supplementary Fig. 2d). It is likely that STAT3 and STAT6, respectively downstream of IL-6 and IL-4, could coordinately act on the regulatory regions of specific target genes^32^. However, as STAT3 acts as a potent transcription activator, such a model appeared insufficient to account for the lack of induction of the targets by IL-6 alone. We therefore considered that there might be additional mechanistic details to IL-6-enhanced IL-4 response in macrophages. Gene ontogeny analyses (via Metascape^33^) was next carried out to probe the function and regulation of the “IL-6-enhanced IL-4 targets”. The biological processes associated with this group of genes showed enrichment in chemotaxis, inflammatory responses and cytokine signaling (Supplementary Fig. 3g). Interestingly, genes associated with regulation of ERK1/2 pathways are also significantly enriched in this group of genes. The M2 phenotype-related chemokine genes, in particular, are within this term. Indeed, both the functions of chemokines and their own inductions are associated with ERK1/2 pathway^34–36^. Therefore, the GO analyses points to an interesting possibility that IL-6-mediated enhancement of IL-4 responses might involve ERK1/2 signaling.

### ERK signaling mediates IL-6-dependent enhancement of IL-4 targets in BMDMs and human macrophages

Given the hints regarding the association of ERK signaling with IL-6-amplified M2 program, we asked whether IL-6 treatment could induce ERK signaling in BMDMs. IL-6 signaling in macrophages is initiated by its binding to the IL-6R receptor subunit, which subsequently activate the signaling receptor subunit gp130^37^. We treated BMDMs with IL-6 or IL-11, the latter being an IL-6-related cytokine that also utilizes gp130 as the signaling receptor^38^. Interestingly, short (30 min) treatments with both cytokines led to visible up-regulation of pERK1/2 levels, in addition to their expected induction of pSTAT3 (Supplementary Fig. 4a). Consistently, IL-11 treatment could also lead to enhancements of IL-4-mediated induction of *Arg1* and *Ccl24* (Supplementary Fig. 4b). Considering such activities by exogenous IL-11, we conducted a control experiment to probe the regulation of endogenous IL-11 under IL-4/IL-6 treatments (Supplementary Fig. 4b, inset). Under the same condition where IL-6 amplified IL-4 responses, no induction of *Il11* mRNA was evident, arguing against a potential secondary involvement of IL-11.

**Figure 3:**
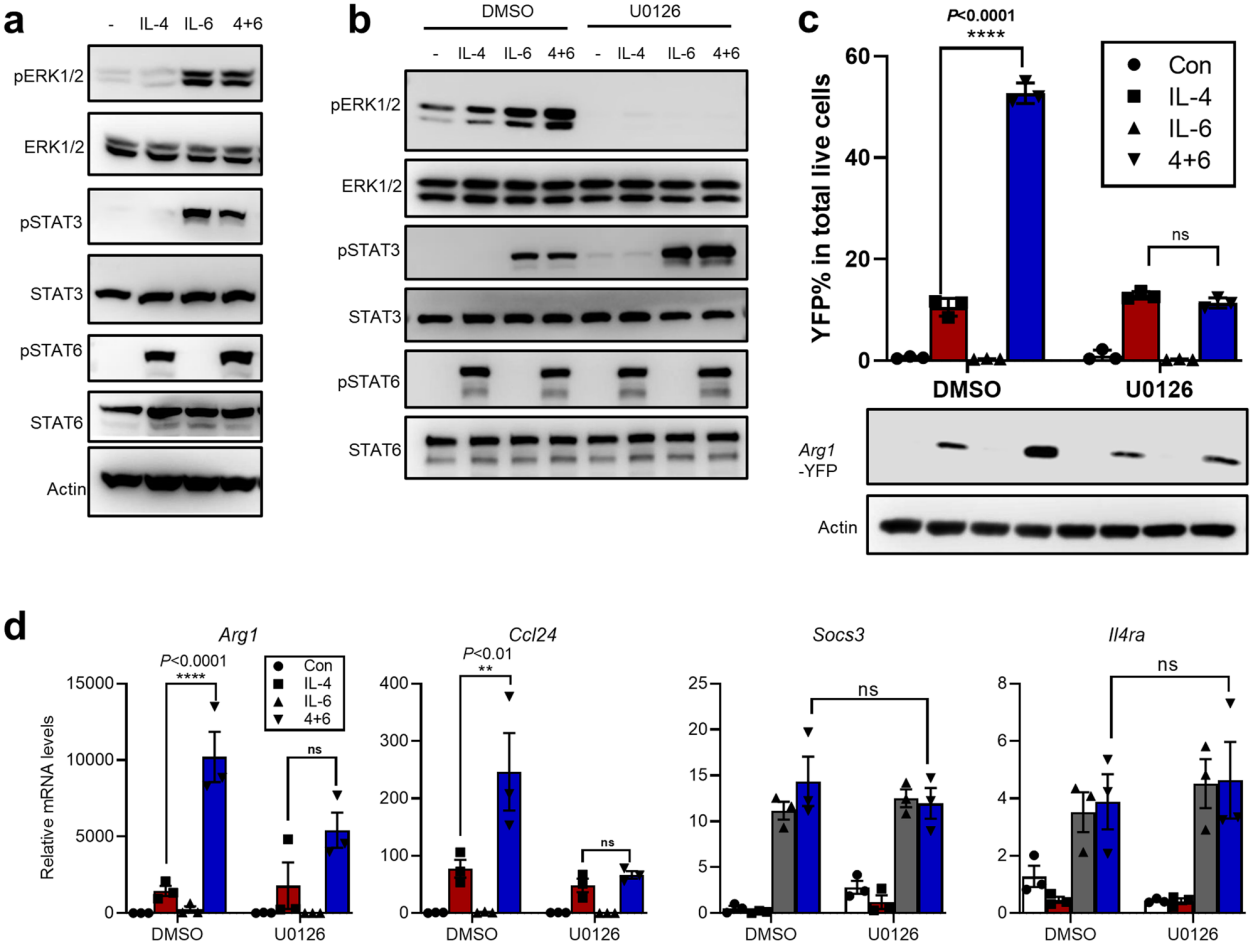
ERK signaling mediates IL-6-dependent enhancement of an IL-4-induced program in macrophages. (**a**) BMDMs were treated with IL-4, IL-6 or IL-4+ IL-6 for 1 h. The cells were harvested and subjected to WB analyses. (**b–d**) BMDMs from *Arg1-YFP* mice were treated with IL-4, IL-6 or IL-4+ IL-6 in the presence or absence of the MEK1/2 specific inhibitor U0126 (1 μM) for 24 h. The cells were harvested and subjected to WB ((**b**) and the lower panel of (**c**)) or for flow cytometry [YFP, n = 3, ± STD] analyses ((**c**), upper panel). The cells were also harvested and subjected to qPCR analyses [n = 3, ± SEM] (**d**). For statistical analyses on different levels of a marker between indicated groups, 2-way ANOVA tests were performed using all data from the given experiment. The adjusted *P* values for specific comparisons are presented on the graphs (****P < 0.0001; **P < 0.01; *ns* not significant).

Next, ERK1/2 signaling was examined in cells treated with IL-4, IL-6 or their combination. Whereas minimal induction of pERK1/2 was seen with IL-4 treatment (1 h), IL-6 and dual stimulation could both promote ERK activation. Consistent with the pattern of pSTAT3 (see also Supplementary Fig. 2c), dual stimulation did not drive further pERK1/2 over the IL-6-only group (Fig. 3a).

To determine the role of ERK signaling in IL-6-amplified M2 program, we used a chemical inhibitor (i.e., U0126^39^) against MEK1/2, the direct upstream kinase for ERK phosphorylation. The BMDMs were treated with IL-4, IL-6 or their combination, in the presence of DMSO (vehicle) or U0126 for 24 h (Fig. 3b). The application of U0126 potently diminished basal and IL-6-enhanced pERK1/2 levels, while causing no inhibition on pSTAT3 (IL-6) or pSTAT6 (IL-4) levels. The genotype of these cells (*Arg1*-YFP) also allowed measurements of *Arg1* levels by YFP fluorescence indexes (Fig. 3c, upper, Supplementary Fig. 4c) and WB (Fig. 3c, lower). Importantly, such analyses showed that targeting ERK pathway in these cells abrogated the enhancement effect of IL-6 on the IL-4-*Arg1* response, without affecting *Arg1* induction by IL-4 alone. The effect by U0126 on other IL-6-amplified M2 target genes mirrored such a pattern (Fig. 3d, Supplementary Fig. 4d). On the other hand, the induction of two known targets of canonical IL-6 responses^25,40^, i.e., *Socs3* and *Il4ra*, under IL-6 or IL-4/IL-6 co-treatment was not altered by U0126. These results strongly suggest the involvement of ERK signaling in IL-6-enhanced M2 response in BMDMs.

In our earlier experiments, pre-treatment/wash-off of IL-6 did not lead to notable enhancement of IL-4 targets (see Supplementary Fig. 2d), suggesting a major role by an on-going IL-6 signaling to establish the crosstalk with IL-4. We therefore probed for IL-6 signaling levels under similar experimental conditions in BMDMs (*Arg1*-YFP). As a positive control, cells were also subjected to short pre-treatment (1 h) of IL-6 without wash-off, followed by IL-4 treatment for 24 h (largely resembling a co-treatment condition). Similar to the earlier patterns on mRNA levels of IL-4 targets (see Supplementary Fig. 2d), the IL-6 pre-treatment/wash-off groups showed minimal indications of remaining ERK1/2 and canonical STAT3 activation, demonstrating attenuation of IL-6 signaling after 24 h of the “chase” period (Supplementary Fig. 4e). In contrast, evident activation of ERK1/2 and canonical STAT3 signaling were observed in the positive control group, correlating to an IL-6-enhanced M2 response reveal by YFP levels (*Arg1*-YFP). Such correlation further corroborated the results from pharmacological experiments (Fig. 3c,d) to support a role of IL-6-ERK signaling in enhancement of M2 responses. We envision that the ERK-dependent axis may cooperate with the previously established IL-6-STAT3 signaling^25,27,31,32^ to empower crosstalk with the IL-4 response.

Next, human PBMC-derived macrophages were treated similarly with IL-4, IL-6 or their combination in the presence of DMSO or U0126. As expected, U0126 selectively inhibited IL-6-mediated increase of pERK1/2 levels (Supplementary Fig. 4f). Given the differences in mouse and human M2 programs^41^, only genes that are known to respond to IL-4 in human macrophages were chosen for analyses^32^. Interestingly, for several human chemokine genes induced by IL-4 (partially overlapping with those in BMDMs, see Supplementary Fig. 3d), an enhancement effect by IL-6 co-addition was evident (Supplementary Fig. 4g). In contrast, IL-6 treatment alone was insufficient to cause their induction. Notably, U0126 prevented the enhancement effect of IL-6 on the response of these genes to IL-4 (Supplementary Fig. 4g). As a control, IL-6-dependent up-regulation of *IL4RA* was not affected (Supplementary Fig. 4h). Collectively, our results in human macrophages also showed that IL-6-ERK signaling contributed to amplifying a group of IL-4 targets.

### An SHP2 inhibitor blocked IL-6-dependent ERK activation and amplification of IL-4 responses

**Figure 4:**
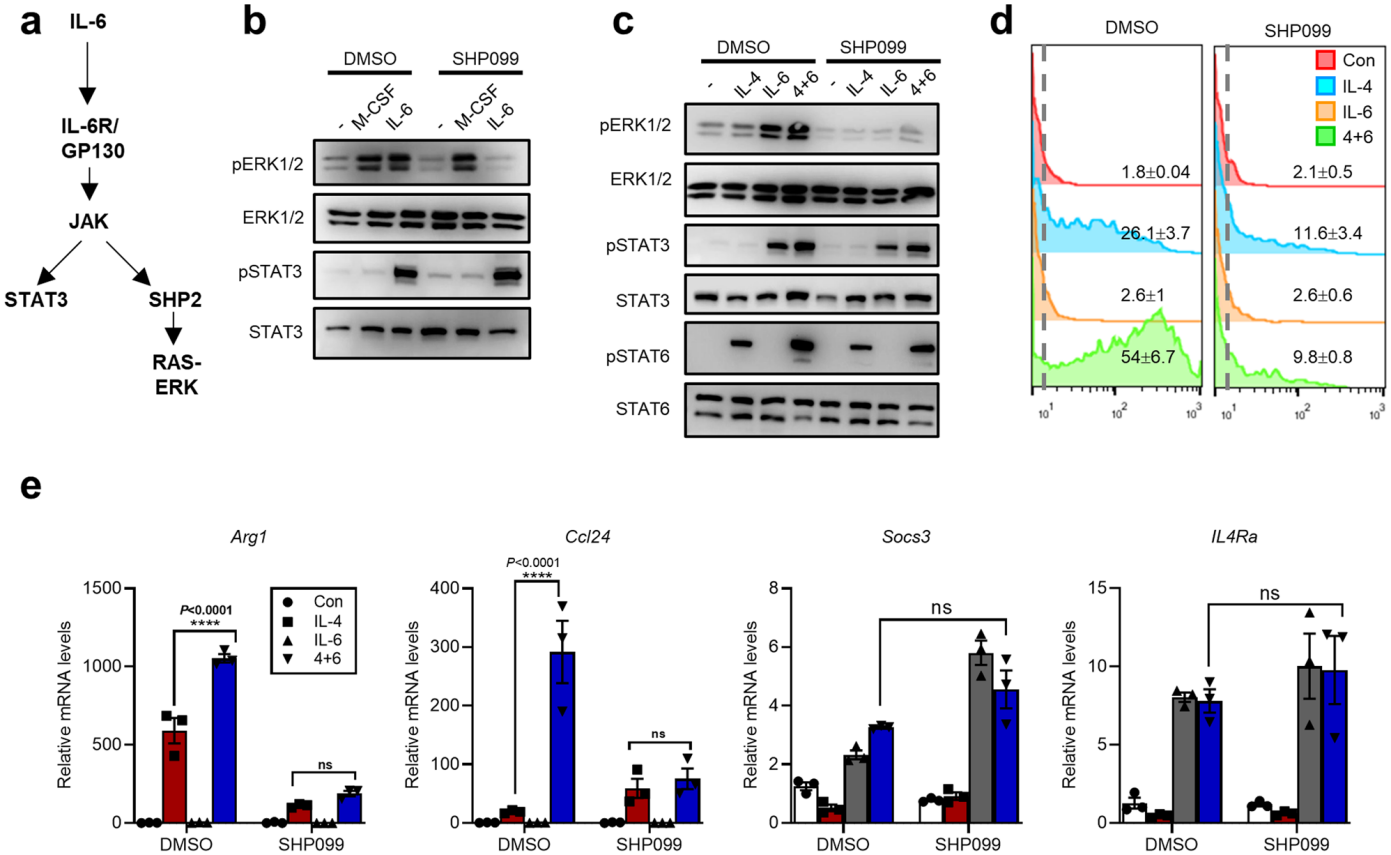
An SHP2 inhibitor blocked IL-6-dependent ERK activation and amplification of IL-4 responses. (**a**) This schematic drawing shows the divergence of canonical STAT3 signaling and SHP2-ERK signaling downstream of IL-6. (**b**) BMDMs were treated with M-CSF (10 ng/ml) or IL-6 (40 ng/ml) for 1 h in the presence of SHP099 (10 μM) or not. The cells were harvested and subjected to WB analyses. (**c–e**) BMDMs from *Arg1-YFP* mice were treated with IL-4 (10 ng/ml), IL-6 (40 ng/ml) or IL-4/IL-6 in the presence or absence of the SHP2 specific inhibitor SHP099 (1 μM) for 24 h. The cells were harvested and subjected to WB analyses (**c**), flow cytometry analyses [YFP, n = 3, ± STDEV] (**d**), or qPCR analyses [n = 3, ± SEM] (**e**). For statistical analyses on different levels of an mRNA between indicated groups, 2-way ANOVA tests were performed using all data from the given experiment. The adjusted *P* values for specific comparisons are presented on the graphs (****P < 0.0001; *ns* not significant).

IL-6-ERK signaling in other systems is driven by an upstream SH2 domain-containing protein tyrosine phosphatase, i.e., SHP2 (Fig. 4a)^37,42^. We therefore next examined the involvement of SHP2 in IL-6-amplified IL-4 response in BMDMs. To this end, we utilized an allosteric SHP2 inhibitor, i.e., SHP099, which stabilizes SHP2 in an autoinhibitory conformation^43^. Expectedly, SHP099 inhibited IL-6-signaled pERK1/2, without affecting pSTAT3 levels (Fig. 4b). In contrast, ERK signaling downstream of M-CSF treatment was not inhibited by blocking SHP2 activity, consistent with a previous report^44^. The BMDMs were next treated with IL-4, IL-6 or their combination in the presence or absence of SHP099. Treatment of SHP099 blocked IL-6-dependent ERK1/2 activation, while causing no inhibition on the levels of pSTAT3 (IL-6) or pSTAT6 (IL-4) (Fig. 4c). Further analyses of *Arg1* levels by YFP (with *Arg1*-YFP BMDMs) showed that pharmacological targeting of SHP2 in these cells abrogated the enhancement effect of IL-6 on the IL-4-*Arg1* response, despite causing a partial inhibition on YFP (*Arg1*) induction by IL-4 alone (Fig. 4d). For the mRNAs levels of *Arg1* and *Ccl24*, SHP099 also largely mitigated the differences between IL-4 and IL4/IL-6 groups, while respectively causing certain levels of inhibition (*Arg1*) and enhancement (*Ccl24*) on induction by IL-4 alone (Fig. 4e). Consistent with unaffected canonical STAT3 signaling (see Fig. 4b,c), the induction of STAT3-associated target genes (*Socs3*^40^ and *Il4ra*^26,45^) by IL-6 or IL-4/IL-6 was not altered by SHP099 (Fig. 4e). Collectively, our results strongly support a critical role of SHP2-ERK axis in mediating IL-6’s enhancement of M2 response in macrophages.

### IL-6-ERK signaling is involved in promoting M2-skewed phenotype of IFN-stimulated transitional monocytes, and potentially in contributing to pro-tumoral polarization of TAMs under poly(I:C) treatment

**Figure 5:**
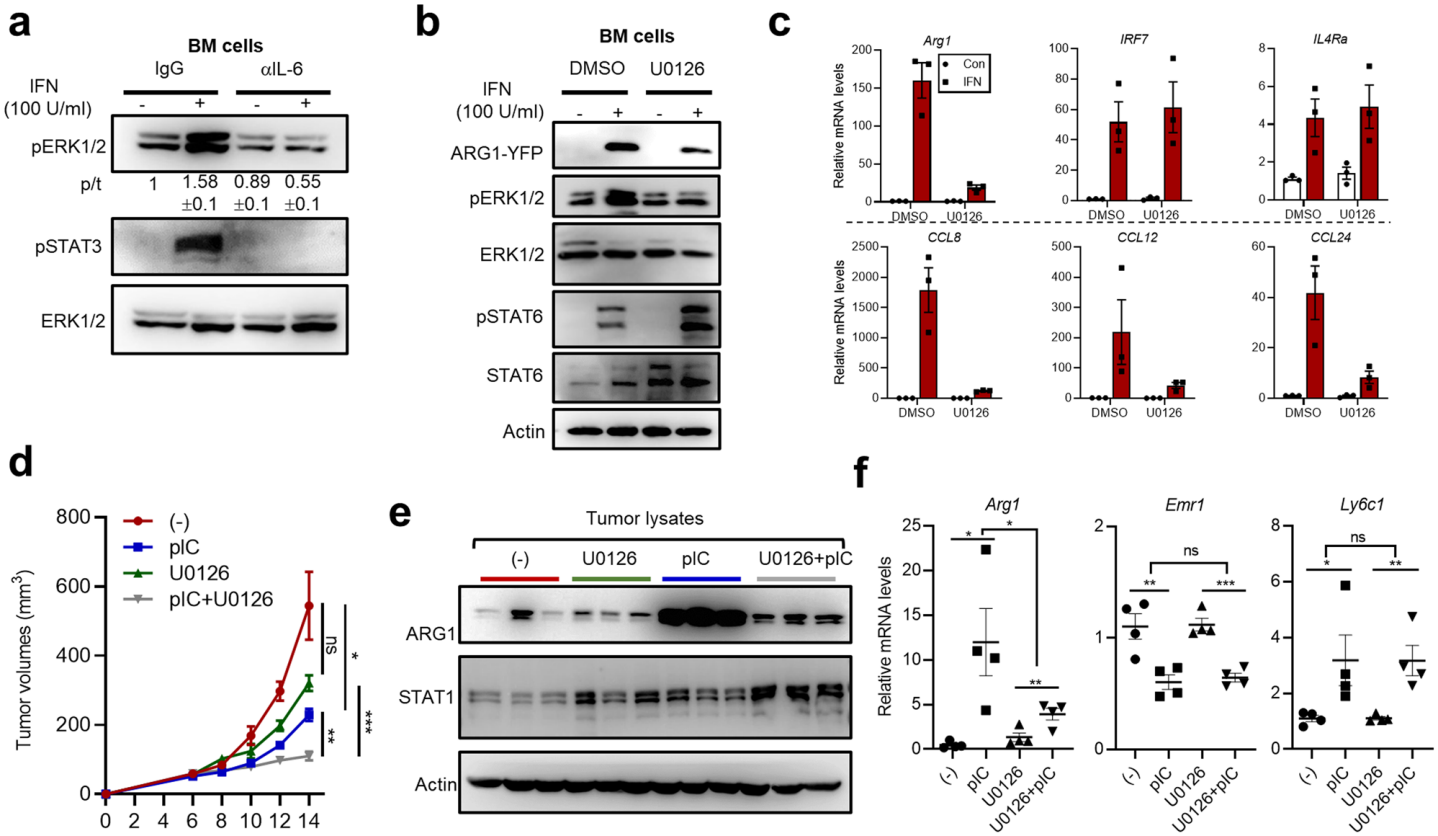
IL-6-ERK signaling is involved in promoting M2-skewed phenotype of IFN-stimulated transitional monocytes, and potentially in contributing to pro-tumoral polarization of TAMs under poly(I:C) treatment. (**a**) BM mononuclear cells were treated with M-SCF (20 ng/ml) ± IFN (100 U/ml) for 24 h, in the presence or absence of IL-6 neutralizing antibody (5 μg/ml). The cells were harvested and subjected to WB analyses. (**b,c**) BM mononuclear cells were treated with M-SCF (20 ng/ml) ± IFN (100 U/ml) for 48 h, in the presence or absence of U0126 (2.5 μM). The cells were harvested and subjected to WB analyses (**b**) or qPCR (n = 3, ± SEM) (**c**). (**d–f**) The mice were injected with 1 × 10^6^ LLC cells in the flanks. Tumor-bearing mice were treated ± poly(I:C) ± U0126 (20 μmol/kg) (i.p.) every 2 days. Tumors were harvested after 4 treatments. In (**d**), the time-dependent changes in tumor sizes are presented (± SEM, n = 4). Multiple unpaired t tests (using Holm–Šídák method) were performed to compare measurements from different groups. The adjusted *P* values for the end-point data were shown on the graph. At the end of the animal experiment, the tumors were harvested. Results from WB (**e**) or qPCR analyses [n = 4, ± SEM] (**f**) are presented. Unpaired t tests were performed for individual markers. Asterisks in this figure correspond to *P* values (***P < 0.001; **P < 0.01; *P < 0.05; *ns* not significant).

In our initial transitional monocytes system that mimic the responses of tumor-associated monocytes/macrophages under IFN-based therapy (Fig. 1), we have demonstrated the involvement of IL-4 and IL-6 in mediating the IFN-elicited, unconventional M2 program. Having established the potential of IL-6-ERK signaling in amplifying IL-4-dependent macrophage response in a series of ligand co-addition experiments (Figs. 3, 4), we next examined whether such a signaling axis also could operate in IFN-stimulated transitional monocytes. Indeed, the neutralizing antibody against IL-6 inhibited IFN-induced pERK1/2 levels in transitional monocytes, correlating to its effect on mitigating pSTAT3 induction (Fig. 5a, and also see Fig. 1h). Indeed, the mRNA levels of IL-6, but not of another IL-6 family member IL-11, were induced in IFN-treated transitional monocytes (Supplementary Fig. 5a). Consistent with the role of IL-6-ERK signaling in promoting macrophage M2 skewing, U0126 treatment significantly reduced IFN-dependent *Arg1*-YFP levels (Fig. 5b), while not affecting pSTAT6 induction (an indication of IL-4 activity, see Supplementary Fig. 1a). Furthermore, U0126 reduced a number of IFN-triggered M2 markers (i.e., *Arg1*, *Ccl8*, *Ccl12* and *Ccl24*), without affecting the induction of *Irf7*, a canonical target of IFN (Fig. 5c). Like the IL-6-treated BMDMs (see Fig. 3d), IFN-stimulated transitional monocytes also showed up-regulation of *Il4ra* mRNA (Fig. 5c), whose levels were not affected by U0126. In addition, U0126 did not significantly alter the patterns of differentiation markers (*Emr1* and *Ly6c1*) respectively associated with macrophages and monocytes (Supplementary Fig. 5b), supporting a specific effect by U0126 in these cells to mitigate their M2-skewed phenotype.

In the transplantable tumor model with Lewis lung carcinoma (LLC), we have previously demonstrated that the IFN-IL-4 cytokine axis-mediated unconventional M2-skewing of TAMs hinders the therapeutic effect by poly(I:C)^21,22^. Given the characterization of IL-6-ERK pathway in amplifying the M2-skewed phenotype of IFN-conditioned transitional monocytes (Fig. 5b,c), it is conceivable that pharmacological targeting of the pathway may potentiate IFN-based therapies. Therefore, we tested the effect of ERK targeting on poly(I:C) treatment of LLC tumors. The mice were administered (i.p.) with ± poly(I:C) and ± U0126 after the tumors became palpable. At the doses administered (20 μmol/kg, every other day), U0126 alone caused modest inhibition of tumor growth, an effect less substantial than that by poly(I:C) alone (Fig. 5d). Notably, combinatory treatment with poly(I:C) and U0126 inhibited tumor growth to a greater degree than by either treatment alone (Fig. 5d). Although U0126 could possibly affect different tumor and non-tumor compartments, these results are in line with the notion that targeting macrophage ERK pathway may enhance poly(I:C) therapy. In the tumor lysates, the direct effects by poly(I:C)/IFN were shown by the increases of STAT1 levels^21,22^ (Fig. 5e). Intriguingly, U0126 alone or in combination with poly(I:C) promoted STAT1 levels, which might be attributed to potential ERK/STAT1 crosstalk previously observed in some cell models^46,47^. As expected from our previous studies^21,22^, poly(I:C) treatment was associated with an induction of ARG1 in the tumors (Fig. 5e). While U0126 treatment alone did not appear to inhibit ARG1 levels in the tumor lysates, its combination with poly(I:C) markedly reduced the treatment-associated ARG1 induction (Fig. 5e). However, our results did not show evident U0126-driven changes in ERK activation at the whole tumor level (Supplementary Fig. 5c), possibly attributing to the insufficient effect by the administered dose of U0126 on the constitutively active RAS-ERK signaling in the LLC tumor cells^48^. In contrast to its strong inhibition of poly(I:C)-engaged *Arg1* expression, U0126 did not significantly alter the whole-tumor expression patterns of *Emr1* and *Ly6c1*, markers respectively associated with macrophages and monocytes (Fig. 5f). As the present treatment model was based on systemic U0126 administration, it is worth pointing out that future detailed analyses of ERK signaling and impacts in different tumor and non-tumor compartments are certainly warranted. Despite such limitations, our data from the above mouse experiments are supportive for a role of macrophage ERK signaling in poly(I:C) treatment-associated, unwanted M2-skewing of TAMs.

## Discussion

IFN-I represents a key component in the innate arm of immune responses against cancer^3,6^. To understand how IFN-I responses are integrated in diverse compartments of tumors shall inspire new strategies to optimize IFN-associated cancer therapies^9^. Here, extending our previous studies on a monocyte-orchestrated IFN-IL-4 cytokine axis that undesirably drive TAMs toward an M2-skewed phenotype^21,22^, we have further identified IL-6 as another mediator for this pro-tumoral axis (Fig. 1). Following induction by IFN, IL-6 acts in concert with IL-4 to augment the levels of a major group of IL-4-dependent target genes in macrophages (Fig. 2, Supplementary Fig. 3). Interestingly, we established a critical role by the IL-6-SHP2-ERK signaling pathway in enhancing the IL-4 response (Figs. 3, 4). Moreover, in the LLC transplantable tumor model, an inhibitor targeting ERK activation mitigated poly(I:C)-induced indexes of M2 TAMs, and leading to further inhibition of tumor growth (Fig. 5). Therefore, our work provides new insights into the tumor-tolerizing role by IFN-I, which shall bear translational implications.

Similar to our previous works^21,22^, the present study was also based on the in vitro culture of M-CSF-instructed transitional monocytes which partially mimic the mixed population of tumor-associated monocytes and macrophages. Together, this series of investigations demonstrate that the IFN-stimulated monocytes may produce multiple cytokines (including IL-4 and IL-6) to subsequently shape downstream responses. The focus of this study is at IL-6, which is a pleotropic cytokine with diverse immunoregulatory, tissue-remodeling and metabolic functions^37,49,50^. IL-6 plays critical roles in host defense, while its dysregulation also drives chronic inflammation and autoimmunity. On the other hand, IL-6 can also contribute to tissue repair and immune tolerance^51^. A prominent example is provided by its known activity to enhance macrophage M2-skewing (in association with restorative activities)^25,27,31,32,52^. Therefore, it is conceivable that a physiological role for the monocyte-mediated IFN-to-IL-4/IL-6 axis may pertain to fine-tuning the balance of immunity and tissue repair under IFN-primed conditions. However, in the tumor context, the response of TAM to such M2-polarizing signals can undesirably drive treatment resistance.

It has been well established that the JAK/STAT3 pathway downstream of IL-6 mediates the enhancement of IL-4 response in macrophages^25,27,31,32^. Our previous results that an STAT3 inhibitor could diminish ARG1 induction in IFN-stimulated transitional monocytes^21^ is in line with this notion, given the clear involvement of endogenous IL-6 and IL-4 in this model (see Fig. 1g, h, Supplementary Fig. 1a). However, the existing mechanistic models for such amplification effect by IL-6 are still unsatisfactory. For instance, a previously suggested STAT3-IL4RA mechanism^25,27^ seems insufficient to account for the fact that some IL-4 target genes are minimally enhanced by IL-6 (see Fig. 2a). Complementing the previous STAT3-centric model^25,27,31,32^, our present results additionally suggest an essential role by the IL-6-ERK1/2 signaling in amplifying the IL-4 response in both mouse and human macrophages (Fig. 3). Moreover, as an upstream signaling component toward ERK activation^37^, SHP2 was also found to be required for the enhancement effect by IL-6 (Fig. 4). It is possible that downstream of IL-6, the ERK-dependent transcription factors such as AP-1 may closely coordinate with STAT3 to facilitate IL-4-activated STAT6 for inducing a subset of M2 genes. Indeed, a role by AP-1 transcription factors in promoting M2 marker gene *Arg1* expression was previously shown^53^. On the other hand, ERK may serve as a direct kinase for serine-727 at STAT3 (in conjunction with canonical signaling via Y705) to enhance its transcriptional activity^54,55^. Interestingly, the S727-phosphorylation of STAT3 has also been shown to increase its translocation to mitochondria and subsequent regulation of OXPHOS^56^. As the phenotypes of M2 macrophages rely on activation of OXPHOS^57^, a potential ERK-STAT3 [S727]-dependent immuno-metabolic mechanism for IL-6/IL-4 crosstalk seem very intriguing. Future works are highly warranted to examine these possibilities and to establish a unified mechanistic model to account for the IL-6-amplied M2 macrophage response.

Our work provides the rationale for combining IFN therapy with ERK inhibition, an intriguing notion considering the established therapeutic implications of ERK pathway in tumors^58^. Although initially considered as a tumor-intrinsic target, recent investigations have also suggested the therapeutic potential of ERK targeting in the immune and non-immune stromal compartments^59^. For instance, ERK activity can drive exhaustive apoptosis of CD8^+^ T cells^60^, and can lead to expansion of TAMs^61^. Compared to the tumor compartment where ERK activities are powered by multiple oncogenic pathways, their activities in the immune compartment may be more susceptible to pharmacological inhibition. Indeed, in our experiments with U0126 (targeting MEK-ERK), an inhibition on the M2 macrophage indexes was already apparent even though global reduction of ERK activity in tumors were not apparent (Fig. 5e,f). Further mechanistic investigations in this and other IFN-related treatment models are warranted to extensively validate such a potential targeting strategy.

Our study also raises some interesting questions. The mechanisms leading to IL-4/IL-6 induction by IFN in monocytes are currently unclear, as these are not considered canonical ISGs. The potential involvement of the unphosphorylated STAT2, as suggested previously^62^, in the induction of such non-canonical ISG warrants testing. It would also be important to explore how our findings may be related to the regulation of human monocytes/TAMs. Interestingly, recent single cell profiling of human TAMs has shown co-existing subsets with signatures associated with IFN and IL-6, or those with regulatory phenotypes resembling M2 macrophages^18,63^. Therefore, the cooperated actions by these cytokines (IFN/IL-6/IL-4) on TAMs might represent a common theme in some human cancers.

Taken together, our study reveals an IFN-triggered IL-4/IL-6 cytokine network impacting on the phenotypes of tumor-associated macrophages. We have provided some further mechanistic insights into IL-6-mediated enhancement of M2 (IL-4) macrophage response. Such investigations may also be relevant for certain pathological conditions involving other IL-6 family members [acting alone or redundantly]^50^. We envision that these findings shall hold therapeutic implications for cancer and other inflammatory diseases.

## Materials and methods

### Ethics statement

The animal studies were approved by the Institutional Animal Care and Use Committee of the Model Animal Research Center of Nanjing University (MARC-NJU) (under project license LJH#19), and were performed in accordance with the relevant guidelines and regulations. The mice were housed in an AAALAC accredited facility (SPF) at MARC-NJU. The experiments involving human PBMCs were approved by the local ethics committee (Nanjing Drum-Tower hospital, Affiliated Hospital of Nanjing University Medical School). Human PBMCs were obtained from healthy volunteers with informed consent at Nanjing Drum-Tower hospital. All methods involving human PBMCs were performed in accordance with the relevant guidelines and regulations.

### Reagents

Polyinosinic–polycytidylic acid (poly(I:C)) was purchased from InvivoGen. Mouse IFNβ (#12405-1) was from PBL. Mouse M-CSF (#315-02), mouse recombinant IL-6 (#315-05), mouse recombinant IL-4 (#214-14), human recombinant M-CSF (#300-25-2), human recombinant IL-6 (#200-06), human recombinant IL-4 (#200-04) were from PeproTech. The ELISA kits for mIL-6 (#88-7064-22) and mIL-4 (#88-7044-22) were from eBioscience.

Mouse IL-4-neutralizing antibodies were from Bio-x cell (#BE0045), mouse IL-6-neutralizing antibody (#504512) was from Biolegend. The ERK1/2 inhibitor U0126 (#U120) was from Sigma. SHP099 (#HY-1003881) was from MCE. The NE-PER Nuclear and Cytoplasmic Extraction kit (#78833) was from Thermo Fisher. Primary antibodies for Western blot were purchased from Cell Signaling Technology (arginase-1, #93668; pSTAT6-Y641, #565543; STAT6, #93623; pStat3-Tyr705, #9145; STAT3, #9139; STAT1, #14994; Erk1/2, #4695; pErk1/2, #4370), and Genscript (Actin, #A00730), Abclonal (GFP, #AE012). Antibodies for Flow Cytometry were purchased form Biolegend: anti-CD16/32 (#101330), AF488-anti-Ly6C (#128022), BV421-anti-F4/80 (#123132), AF647-anti-CD206 (#141712).

### Flow cytometry analyses

For analysis of YFP (*Arg1-YFP*) levels, BMDMs were dissociated from the culture plate using 5 mM EDTA in PBS. Following washing with PBS, the prepared cells were directly subjected to flow cytometry analysis. FACS was also applied for separation of monocytes and macrophages. After 2 days treatment with M-CSF ± IFNβ (100 U/ml), the monocytes (Ly6C^+^F4/80^−^) and macrophages (F4/80^+^) were sorted with a BD FACSAria III high-speed sorter. To analyze the expression of CD206, an intracellular staining protocol was adopted according to the instruction from the antibody provider (Biolegend). Cells were fixed and permeabilized using BD Cytofix/Cytoperm Plus (#554715, BD), and subsequently subjected to antibody staining for 30 min.

### Differentiation of mononuclear cells

Mouse bone marrow was harvested from *Arg1-YFP*^+/+^ mice^22^ at the age of 8 to 12 weeks. The cut femur and tibia were flushed with PBS with the use of 25-gauge needles. Then the cell suspensions were passed through a 40-μm nylon mesh. The BM mononuclear cells were prepared by Histopaque-1077 gradient centrifugation (#17-5442-02, GE Healthcare)^21^. These cells were then cultured with DMEM medium (#10564029, Gibco) containing 10% FBS (Gibco) and 20 ng/ml of recombinant mouse M-CSF. BM-derived cells were cultured for a total of 7 days (fed with fresh medium containing M-CSF every other day) to generate mature macrophages. Human PBMCs were prepared via a similar gradient centrifugation protocol as above. For derivation of human macrophages, PBMCs were cultured for 7 days in RPMI-1640 medium containing 10% FBS (Gibco) and human M-CSF (20 ng/ml).

### Mouse tumor model

The C57/BL6 mice (6–8 weeks) were injected subcutaneously (s.c.) with 1 × 10^6^ Lewis lung carcinoma (LLC) cells at the flanks^22^. The tumors growth was monitored and measured ([length x width^2^]/2). Treatments generally started on day 6 when tumor volumes reached ~ 50 mm^3^, and lasted for 8 days. Poly(I:C) (5 mg/kg, i.p.) or U0126 (20 μmol/kg^64^, i.p.) was administered every two days. The animal study is reported in accordance with ARRIVE guidelines.

### Western blot and quantitative PCR analyses

Western blot analyses were performed as previously described^21^. In some experiments, cytosolic or nuclear extracts were first prepared with a kit from Thermo according to manufacturer’s instructions. Enhanced chemiluminescence (ECL) was used for detection. Total RNA of cells was isolated utilizing the Trizol reagents. Equal amounts of RNA were then subjected to reverse transcription using HiScript Q RT SuperMix (#R123-01, Vazyme). Primers used are shown in the Supplementary Table 1. Quantitative PCR (qPCR) analyses were performed on ABI Step One Plus using SYBR premix (#Q141-AA, Vazyme) or Roche LightCycler (#Q121-AA, Vazyme). The housekeeping gene GAPDH was used as internal control.

### RNA sequencing

BMDMs treated with IL-4 (10 ng), IL-4 (10 ng) + IL-6 (40 ng) for 24 h, all cells were harvested and lysed in Trizol reagent. The RNA sequencing procedure and data analyses were carried out by Annoroad Gene Tech. Co., Ltd. (Beijing, China).

### Statistical analysis

Statistical analysis was performed using GraphPad Prism. All data presented in this study are derived from at least three independent experiments. For quantitative results, average values from biological replicates were presented with error bars denoting SEM (n ≥ 3). Depending on the experimental designs, the *P* values were determined by Student t-tests, multiple t tests [using Holm-Šídák method], or 2-way ANOVA as indicated in the figure legends (*P < 0.05, **P < 0.01, ***P < 0.001, ****P < 0.0001).

## Supplementary Information

Supplementary Information.

## Data Availability

High-throughput RNAseq data (raw and processed) are deposited at the NCBI GEO database (https://ncbi.nlm.nih.gov/geo/query/acc.cgi?acc=GSE192371). The following secure token has been created: wfcvakguntovlwp.
