# Supplementary material for: IL-6/ERK signaling pathway participates in type I IFN-programmed, unconventional M2-like macrophage polarization
Source: Sci Rep. 2023 Feb 1;13:1827. doi: 10.1038/s41598-022-23721-9 (PMC9892596; doi:10.1038/s41598-022-23721-9)
Supplement: Supplementary file 1 — Supplementary Information. [file 41598_2022_23721_MOESM1_ESM.docx]

**Supplementary Information**

**IL-6/ERK signaling pathway participates in type I IFN-programmed, unconventional M2-like macrophage polarization**

- **Supplementary Figures and Legends**
- **Supplementary Table 1:** Primers for qPCR analyses
- **Raw WB blots corresponding to data in the Figures and Supplementary Figures**

**Supplementary Figures and Legends:**

**Supplementary Figure 1: IFN-stimulated transitional monocytes release soluble factors for induction of ARG1 in macrophages**. **(a)** BM mononuclear cells from *Arg1-YFP* mice (with an illustration of the modified allele) were treated with M-SCF (20 ng/ml) ± IFN (100 U/ml) for 48 h with or without the presence of antibodies against IL-4 (5 μg/ml). Samples were subjected to WB or qPCR analysis (n =3, ±SEM). **(b-d)** BMDMs from *Arg1-YFP* mice were stimulated with different doses of IL-4 for 24 h. (b) Cells were harvested for flow cytometry (YFP signals). In (c), the relative fluorescent levels [YFP%×MFI] for YFP were quantitated (n =3, ±SEM). (d) Cells were also harvested for WB analyses. **(e)** BM mononuclear cells were treated with M-CSF ± IFNβ for 48 h. In some groups, neutralizing Abs against IL-10 (5 μg/ml) were added to the medium as indicated. The cells were harvested and subjected to qPCR analyses (n =3, ±SEM). **(f)** BM mononuclear cells were treated with M-SCF ± IFN for 48 h, monocytes (Ly6C^+^F4/80^-^) and macrophages (F4/80^+^) were sorted by fluorescent cytometry. The RNA samples from sorted cells were analyzed by qPCR for *Il4* (n =3, ±SEM). For statistical analyses on different levels of an mRNA between indicated groups, 2-way ANOVA tests were performed using all data from the given experiment. The adjusted *P* values for specific comparisons are presented on the graphs (****: *P*<0.0001; ***: *P*<0.001; ns: not significant).

**Supplementary Figure 2: IL-6 signaling can tightly coordinate with IL-4 or IL-13 signaling to enhance the M2 macrophage response. (a-b)** BMDMs from *Arg1-*YFP mice were either untreated or treated with IL-4 (10 ng/ml), IL-6 (40 ng/ml), IL-13 (40 ng/ml), IL-4 (10 ng/ml) +IL-6 (40 ng/ml), or IL-6 (40 ng/ml) +IL-13 (40 ng/ml) for 24 h. The cells were harvested and subjected to qPCR [n =3, ±SEM] (a) or for flow cytometry (YFP%) analyses [n =3, ±STD] (b). The qPCR results were also subjected to statistical analyses (unpaired t tests) on the differences between indicated groups. The *P* values are presented on the graphs (**: *P*<0.01). **(c)** Phosphorylated STAT3 and STAT6 levels in cytoplasmic and nuclear extracts from the indicated cytokine-treated BMDMs for 30 min are shown. **(d)** BMDMs were pre-incubated with IL-6 (40 ng/ml) or medium alone for 24 h. After 24 h, cells were washed and stimulated with ±IL-4 (10 ng/ml) for another 24 h. Another group was subjected to simultaneous IL-4 and IL-6 treatment for 24 h (blue). Samples were analyzed by qPCR (n =3, ±SEM).

**Supplementary Figure 3: Co-addition of IL-6 with IL-4 leads to enhanced expression in a major group of IL-4 targets, together with other patterns of regulation of gene expression. (a-d)** BMDMs were treated with IL-4 (10 ng/ml), or IL-4 (10 ng/ml) + IL-6 (40 ng/ml) for 24 h. The RNAseq analyses were performed. Principal-component analysis (PCA) of gene expression data is shown in (a). Each dot represents a biological replicate for an indicated treatment group. The first principal component (PC) is shown on the X-axis, and the second PC is shown on the Y-axis. Numbers of induced genes upon IL-4 (10 ng/ml) or IL-4 (10 ng/ml) +IL-6 (40 ng/ml) stimulation are presented in (b). The number of identical genes within the two groups and that of the combined gene list are also shown in the graph. Genes that featured induction in either the IL-4- and/or the co-addition-group (Adjusted *P*<0.05, Log_2_FC>2) were clustered, with the heatmap presented in (c). Furthermore, the IL-6-amplified (Log_2_FC>1.5) M2 genes (induced by IL-4) were selected, with the heatmap presented in (d). **(e)** BMDMs were treated with IL-4, IL-6 or IL-4/IL-6 for 24 h, indicated mRNAs were analyzed by qPCR (n =3, ±SEM). The qPCR results were also subjected to statistical analyses (multiple unpaired t tests, using Holm-Šídák method) for the differences between indicated groups. The adjusted *P* values are presented on the graphs (***: *P*<0.001; **: *P*<0.01; *: *P*<0.05; ns: not significant). **(f)** The aggregated expression levels (FKMP) for two group of genes under the condition of IL-4 treatment are shown (left, IL-6-enhanced M2 genes; right, IL-6-suppressed M2 genes). The *P* value for unpaired t test is presented. **(g)** List of top 20 significantly enriched GO biological processes for IL-6 amplified IL-4 target genes in BMDMs. Those genes associated with ERK pathways are listed.

**Supplementary Figure 4: ERK signaling mediates IL-6-dependent enhancement of IL-4 targets in macrophages. (a)** BMDMs were treated with IL-6 (40 ng/ml) or IL-11 (40 ng/ml) for 1 h. The cells were harvested and subjected to WB analyses. **(b)** BMDMs were either untreated or treated with IL-4 (10 ng/ml), IL-6 (40 ng/ml), IL-11 (40 ng/ml), IL-4 (10 ng/ml) +IL-6 (40 ng/ml) together, or IL-4 (10 ng/ml) +IL-11 (40 ng/ml) for 24 h. The cells were harvested and subjected to qPCR (n =3, ±SEM). The mRNA levels of *Il11* in single and combinatorial IL-4/IL-6 treatment groups are shown in the inset (n =3, ±SEM). **(c-d)** BMDMs from *Arg1-YFP* mice were treated with IL-4 (10 ng/ml), IL-6 (40 ng/ml) or IL-4 (10 ng/ml) +IL-6 (40 ng/ml) in the presence or absence of the ERK1/2 specific inhibitor U0126 (1 μM) for 24 h. The cells were harvested and subjected to fluorescent cytometry [YFP, n =3, ±STD] (c)**,** or qPCR analyses [n =3, ±SEM] (d). **(e)** BMDMs were pre-incubated with IL-6 (40 ng/ml) or medium alone for 24 h (“D1”). After 24 h, cells were washed and stimulated with ±IL-4 (10 ng/ml) for another 24 h (“D2”). Another group was subjected to short IL-6 pre-treatment without wash-off, followed by IL-4 treatment for 24 h [abbreviated as “(6*+4) D2” with the “6*” denoting short IL-6 pre-treatment]. Samples were analyzed by WB. **(f-g)** Human macrophages (hMDMs) were treated with IL-4 (20 ng/ml), IL-6 (20 ng/ml) or IL-4 (20 ng/ml) + IL-6 (20 ng/ml), in the presence or absence of the ERK specific inhibitor U0126 (1 μM) for 24 h. The cells were harvested and subjected to WB (f) or qPCR analyses [n =3, ±SEM] (g, h). For statistical analyses on different levels of an mRNA between indicated groups, 2-way ANOVA tests were performed using all data from the given experiment. The adjusted *P* values for specific comparisons are presented on the graphs (****: *P*<0.0001; **: *P*<0.01; *: *P*<0.05; ns: not significant).

**Supplementary Figure 5: U0126 did not significantly affect the differentiation markers of monocytes and macrophages in vitro. (a)** BM mononuclear cells were treated with M-SCF (20 ng/ml) ± IFN (100 U/ml) for 48 h. The mRNA levels of *Il6* and *Il11* were determined by qPCR (n =3, ±SEM). **(b)** BM mononuclear cells were treated with M-SCF (20 ng/ml) ± IFN (100 U/ml) for 48 h, in the presence or absence of U0126 (2.5 μM). The cells were harvested and subjected to qPCR analyses (n =3, ±SEM). **(c)** Tumor lysates from indicated experimental group were analyzed by WB. Densitometry analyses for bands of pERK1/2 or actin were performed for quantitation.

**Supplementary Table 1**: Primers for qPCR analyses

| **Symbol (mouse)** | **Forward primer sequences (5’-3’)** | **Reverse primer sequences (5’-3’)** |
| --- | --- | --- |
| *Irf7* | CACAGATCTTCAAGGCCTGGGC | CTGTGGAGTGCACAGCGGAAGT |
| *Arg1* | TTGGGTGGATGCTCACACTG | TTGCCCATGCAGATTCCC |
| *Fizz1* | TACTTGCAACTGCCTGTGCTTACT | TATCAAAGCTGGGTTCTCCACCTC |
| *Ym1* | TGGAGGATGGAAGTTTGGAC | AATGATTCCTGCTCCTGTGG |
| *Il4* | GGTCTCAACCCCCAGCTAGT | GCCGATGATCTCTCTCAAGTGAT |
| *Il6* | TAGTCCTTCCTACCCCAATTTCC | TTGGTCCTTAGCCACTCCTTC |
| *Il10* | GCTCTTACTGACTGGCATGAG | CGCAGCTCTAGGAGCATGTG |
| *Il11* | TGTTCTCCTAACCCGATCCCT | CAGGAAGCTGCAAAGATCCCA |
| *Il4ra* | TCTGCATCCCGTTGTTTTGC | GCACCTGTGCATCCTGAATG |
| *Ccl2* | GGCTCAGCCAGATGCAGTTAA | CCTACTCATTGGGATCATCTTGCT |
| *Ccl8* | TCTACGCAGTGCTTCTTTGCC | AAGGGGGATCTTCAGCTTTAGTA |
| *Ccl12* | ATTTCCACACTTCTATGCCTCCT | ATCCAGTATGGTCCTGAAGATCA |
| *Ccl17* | GACGACAGAAGGGTACGGC | GCATCTGAAGTGACCTCATGGTA |
| *Ccl24* | TCTTGCTGCACGTCCTTTATT | GCATCCAGTTTTTGTATGTGCC |
| *Mrc1* | CTCTGTTCAGCTATTGGACGC | CGGAATTTCTGGGATTCAGCTTC |
| *Chil4* | TCCACTTTGAACCACATTCCAA | CCAGCACTAACAGTAGGGTCA |
| *Socs3* | ATGGTCACCCACAGCAAGTTT | TCCAGTAGAATCCGCTCTCCT |
| *Emr1* | CCCCAGTGTCCTTACAGAGTG | GTGCCCAGAGTGGATGTCT |
| *Ly6c1* | GCAGTGCTACGAGTGCTATGG | ACTGACGGGTCTTTAGTTTCCTT |
| *Gapdh* | AGGGCTGCTTTTAACTCTGGT | CCCCACTTGATTTTGGAGGGA |
|  |  |  |
| **Symbol (human)** | **Forward primer sequences (5’-3’)** | **Reverse primer sequences (5’-3’)** |
| *CCL8* | TGGAGAGCTACACAAGAATCACC | TGGTCCAGATGCTTCATGGAA |
| *CCL17* | CATCTGAGGACTGCTCCAG | GCTTTGCAGGTATTTAACTGC |
| *CCL18* | GGTGTCATCCTCCTAACCA | GTCGCTGATGTATTTCTGGAC |
| *CCL23* | CATCTCCTACACCCCACGAAG | GGGTTGGCACAGAAACGTC |
| *IL4Ra* | *CGTGGTCAGTGCGGATAACTA* | *TGGTGTGAACTGTCAGGTTTC* |
| *GAPDH* | AGGGCTGCTTTTAACTCTGGT | CCCCACTTGATTTTGGAGGGA |

**Raw WB blots corresponding to data in the Figures and Supplementary Figures:**
